# Peptidyl-prolyl isomerase-B is involved in *Mycobacterium tuberculosis* biofilm formation and a generic target for drug repurposing-based intervention

**DOI:** 10.1038/s41522-018-0075-0

**Published:** 2019-01-15

**Authors:** Ashutosh Kumar, Anwar Alam, Sonam Grover, Saurabh Pandey, Deeksha Tripathi, Monika Kumari, Mamta Rani, Aditi Singh, Yusuf Akhter, Nasreen Z. Ehtesham, Seyed E. Hasnain

**Affiliations:** 1 JH-Institute of Molecular Medicine, Jamia Hamdard, New Delhi, India; 2 Molecular Infection and Functional Biology Lab, Kusuma School of Biological Sciences, Indian Institute of Technology-Delhi, New Delhi, India; 3 National Institute of Pathology, Safdarjung Hospital Campus, New Delhi, India; 4 Department of Microbiology, Central University of Rajasthan, Ajmer, Rajasthan India; 5 Centre for Computational Biology and Bioinformatics, School of Life Sciences, Central University of Himachal Pradesh, Himachal Pradesh, India; 6 Department of Biochemical Engineering and Biotechnology, Indian Institute of Technology-Delhi, New Delhi, India; 7 School of Biotechnology, Jawaharlal Nehru University, New Delhi, India; 8 Department of Biotechnology, Babasaheb Bhimrao Ambedkar University, Lucknow, India; 9 Dr Reddy’s Institute of Life Sciences, University of Hyderabad Campus, Prof CR Rao Road, Hyderabad, India; 10 Present Address: Department of Microbiology, Tripura Central University, Suryamaninagar, Agartala, Tripura India; 11 Present Address: Department of Biochemistry, Jamia Hamdard, New Delhi, India

## Abstract

Tuberculosis (TB), a disease caused by *Mycobacterium tuberculosis* (*M.tb*), takes one human life every 15 s globally. Disease relapse occurs due to incomplete clearance of the pathogen and reactivation of the antibiotic tolerant bacilli. *M.tb*, like other bacterial pathogens, creates an ecosystem of biofilm formed by several proteins including the cyclophilins. We show that the *M.tb* cyclophilin peptidyl-prolyl isomerase (PpiB), an essential gene, is involved in biofilm formation and tolerance to anti-mycobacterial drugs. We predicted interaction between PpiB and US FDA approved drugs (cyclosporine-A and acarbose) by in-silico docking studies and this was confirmed by surface plasmon resonance (SPR) spectroscopy. While all these drugs inhibited growth of *Mycobacterium smegmatis* (*M.smegmatis)* when cultured in vitro, acarbose and cyclosporine-A showed bacteriostatic effect while gallium nanoparticle (GaNP) exhibited bactericidal effect. Cyclosporine-A and GaNP additionally disrupted *M.tb* H_37_Rv biofilm formation. Co-culturing *M.tb* in their presence resulted in significant (2–4 fold) decrease in dosage of anti-tubercular drugs- isoniazid and ethambutol. Comparison of the cyclosporine-A and acarbose binding sites in PpiB homologues of other biofilm forming infectious pathogens revealed that these have largely remained unaltered across bacterial species. Targeting bacterial biofilms could be a generic strategy for intervention against bacterial pathogens.

## Biofilms: Repurposing drugs against tuberculosis

Tuberculosis, caused by *Mycobacterium tuberculosis*, is the leading cause of death due to a single infectious agent. New therapeutic options are needed, and repurposing clinically approved drugs to destroy biofilms is an attractive approach, as these microbial communities are often less susceptible to antibiotics. A team lead by Seyed Hasnain at the Indian Institute of Technology Delhi identified an enzyme, PpiB, from *M. tuberculosis* that promoted biofilm formation and showed that PpiB interacts with several drugs that are currently used to treat diabetes, immunological diseases and cancer. These drugs destabilise *M. tuberculosis* biofilms in culture and enhanced the potency of two current anti-tuberculosis antibiotics. Future work is needed to test these medications against tuberculosis in humans, but given PpiB is found in different bacteria, there may be broader promise of using these repurposed drugs to combat other infections.

## Introduction

Biofilm associated diseases cause nearly 80% of the recalcitrant hospital infections.^1^ Several non-pathogenic and pathogenic species of microorganism including mycobacteria make biofilm as one of the generic mechanisms to overcome stress. The matrix of the biofilm is composed of extracellular components consisting of biopolymers that are essentially secreted by the microorganisms and act as a physical barrier to drugs or against immune surveillance. In addition to the rapid emergence of drug resistance in several strains of mycobacteria, the growing menace of drug tolerance has led to the requirement of higher doses of drugs for effective management of diseases such as tuberculosis (TB).^2^ There is acute shortage of drugs that can be used against biofilm forming pathogens. The inherent ability of the pathogen to evolve under selective drug pressure outpaces the rate of development of new drugs, and diminishes the efficacy of the drug by the time it is commercially available. The eminent solution is to expedite new arsenal of drugs against classical and non-classical targets in *M.tb* proteome or establish new roles for currently available drugs. Drugs licensed for other known disorders in humans related to mental illness, diabetes, malaria etc. target cellular pathways which are also utilised by *M.tb* for survival. Drug repurposing offers a viable option to fast track new therapies against other diseases.^3^

Previous studies showed the involvement of biofilm formation by *Mycobacterium abscessus* and *Pseudomonas aeruginosa* in cystic fibrosis and also as a virulence determinant in uropathogenic *Escherichia coli* isolates.^4–6^ The presence of extracellular *M.tb* within biofilm like structure inside lung lesions of *M.tb* infected guinea pigs undergoing antibiotic treatment points to the possibility of biofilm formation within the host tissues.^7^ Peptidyl-prolyl isomerases (PPIase), popularly known as cyclophilins, are ubiquitously expressed protein foldases which aid in protein folding or refolding by accelerating the rate-limiting cis-trans and trans-cis-conformational changes at Xaa-Pro bonds.^8^ *M.tb* Ppiases is also involved in chaperonic activity, chromatin remodelling, regulatory processes in the cell, RNA-mediated gene expression, modulating of infections etc.^9,10^ Several FDA approved drugs and nanoparticle based therapies are being repurposed against biofilms and have shown promising results. Anti-helminth drug, niclosamide, has shown inhibitory effects against biofilms formed by *P.aeruginosa*.^11^ Nanoparticles, by virtue of their small size and charge have also been effective as antimicrobial agents. Silver nanoparticles have shown promising results as an alternative agent to inhibit bacterial biofilms.^12^

The biology of *M.tb* biofilm formation and its clinical relevance is scant in the literature. Pellicles formed at the liquid–air interface of a static culture are working model for in vitro studies on biofilms.^13^ In the present study we elucidate the role of *M.tb* PpiB and identify drug repurposing-based biofilm inhibitors. Recombinant *M.smegmatis* cells carrying *M.tb* PpiB gene under anhydrotetracycline inducible promoter, was used as a model for biofilm studies. We show that heterologous expression of *M.tb* PpiB in *M.smegmatis* exhibited enhanced biofilm formation as compared to wild type *M.smegmatis*, pointing to its likely role in developing drug tolerance. Previous studies^14^ pointed to the possible interaction of PpiB with cyclosporine-A rendering it a possible candidate among US FDA approved drug for inhibition of biofilms. Recent reports suggest that gallium, a FDA approved agent used in cancer related hypercalcemia and cancer diagnostics, has been repurposed for antimicrobial therapies.^15,16^ In-silico studies supported by SPR data showed that acarbose, a FDA approved drug against diabetes, and cyclosporine-A, a FDA approved immunosuppressant used in patients undergoing organ transplantation, interact with PpiB and inhibit biofilm forming activity of PpiB. A comparison of PpiB homologues in different groups of biofilm forming pathogens reveals that the binding residues that interact with cyclosporine-A or acarbose have largely remained conserved, thereby pointing to its efficacy as a putative candidate for targeting biofilms across a wide genre of microorganisms. To our knowledge, the present study proves that PpiB is a suitable candidate to target biofilm forming organisms. We also demonstrate that cyclosporine-A, acarbose or GaNP can reduce the dosage of anti-TB drugs and can be used as conjunct drug/agent for targeting biofilm associated diseases involving other bacteria.

## Results

### Recombinant *M.smegmatis* expressing *M.tb* PpiB show increased biofilm formation in vitro

**Fig. 1:**
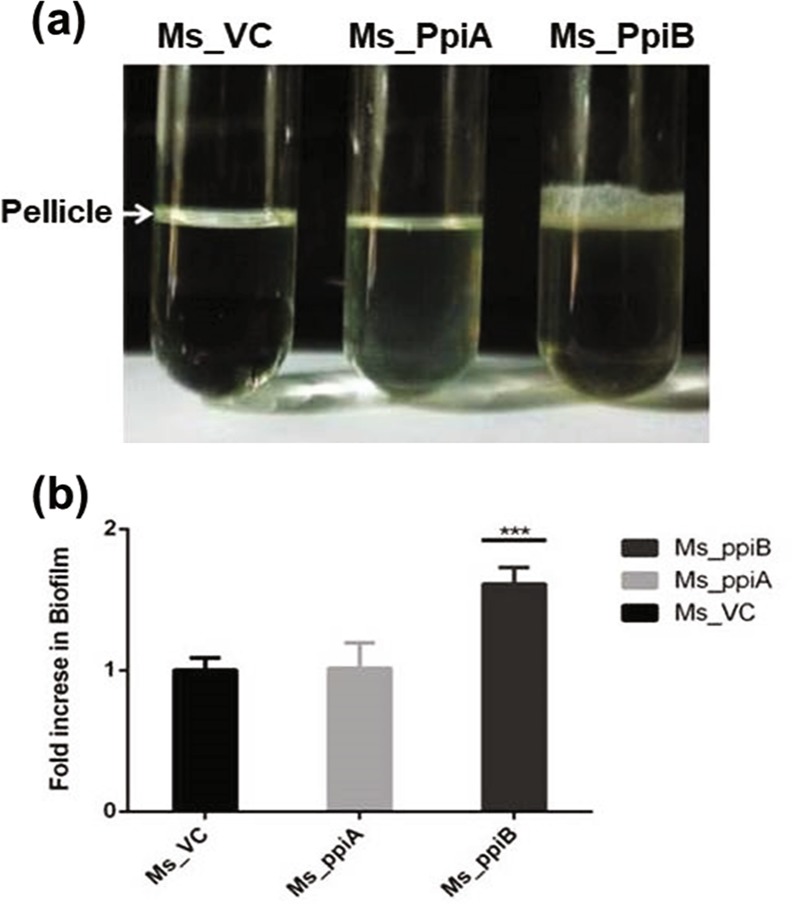
*M.tb* PpiB protein enhances biofilm and pellicle formation in recombinant *M.smegmatis*: *M. tb* PpiA and PpiB genes were cloned in *M.smegmatis*, as described in methods, to generate Ms_PpiA and Ms_PpiB strains, respectively. *M.smegmatis* vector control (Ms_VC), Ms_PpiA and Ms_PpiB were grown in static culture and induced with anhydrotetracycline for expressing *M.tb* PPIases. **a** Pellicle formed in Ms_VC, Ms_PpiA and Ms_PpiB cultures from a representative experiment. The pellicle formed at the liquid air interface was quantified as described in methods. Values shown in **b** are mean [±s.e.m] from three independent experiments and depict the amount of biofilm formed in Ms_VC, Ms_PpiA and Ms_PpiB cultures. ***p < 0.005 vs. Ms_VC (Student’s *t*-test)

*M.smegmatis* vector control (Ms_VC) lacking either *M.tb*_PpiA or *M.tb*_PpiB genes were used as control to examine the role of *M.tb* PpiA and PpiB in biofilm formation. Ms_VC and recombinant *M.smegmatis* (Ms_PpiA and Ms_PpiB) were induced by culturing cells in the absence and presence of anhydrotetracycline, as described in methods. Results in Fig. 1a show that Ms_VC and Ms_PpiA express basal level of biofilm, indicating that *M.tb* PpiA gene does not contribute to biofilm formation. It could be seen (Fig. 1b) that Ms_PpiB exhibited nearly 1.5-fold increase (*p* < 0.005) in biofilm formation as compared to either Ms_VC or Ms_PpiA cells. These results demonstrate the involvement of *M.tb* PpiB in biofilm formation, thereby modulating the cell surface properties of the pathogen.

### Cyclosporine-A, acarbose, or GaNP binding sites in PpiB homologues in biofilm forming bacterial species have largely remained unaltered; Evidence of physical interaction

**Fig. 2:**
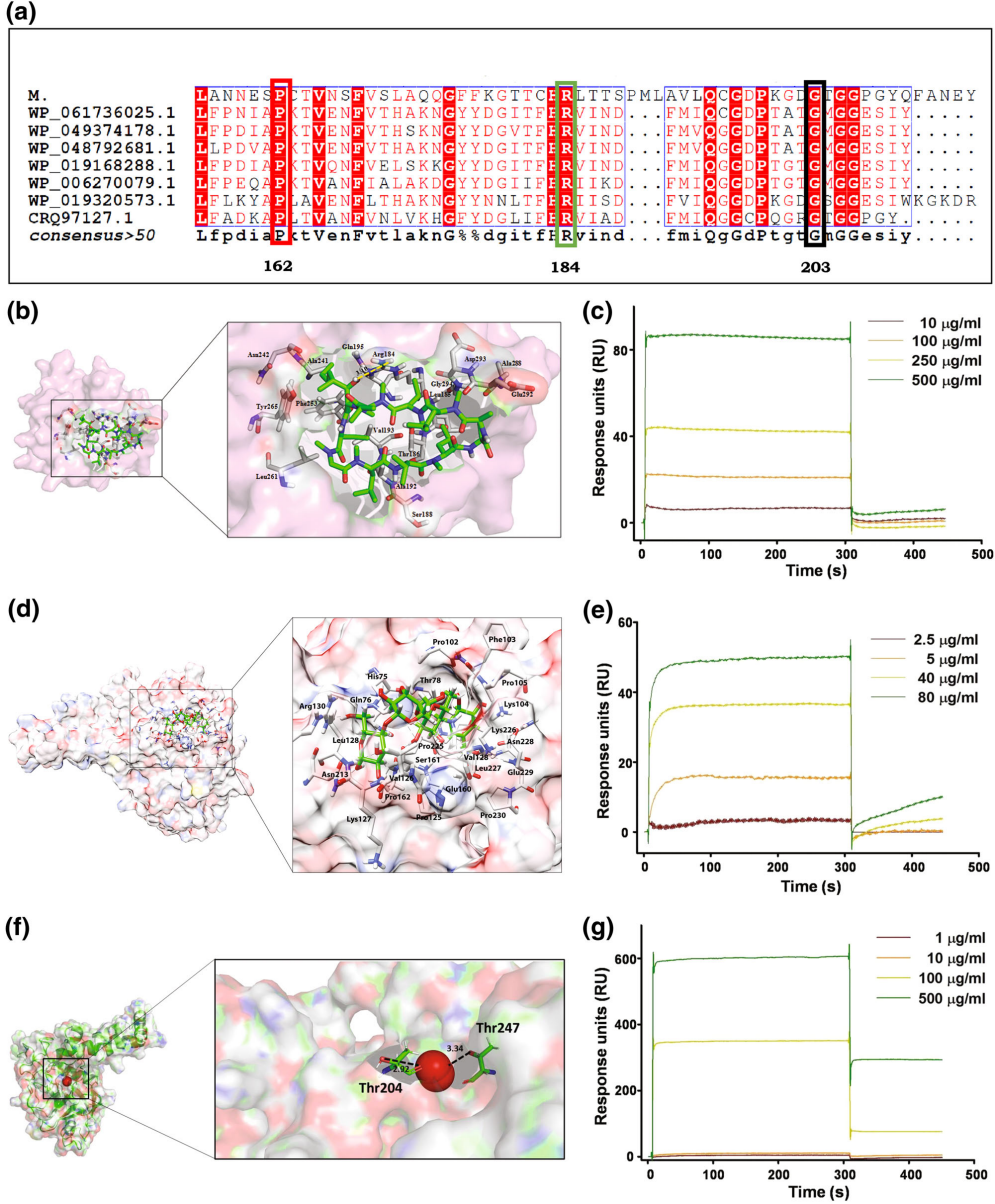
Multiple sequence alignment of *M.tb* PpiB in biofilm forming bacteria and interaction of PpiB with cyclosporine-A, acarbose and GaNP. **a** *M.tb* PpiB (Rv2582) exhibits homology with proteins from other biofilm forming bacteria and possesses similar amino acids Arg and Pro at the binding site of cyclosporine-A (highlighted in green box) and acarbose (highlighted in red box), respectively. A dimer of atomic gallium^17^ similarly binds to Gly residue (highlighted in black box), which is conserved within the PpiB binding site of all biofilm-forming bacteria. **b**, **d**, **f** Interaction of cyclosporine-A, acarbose and dimer of atomic gallium with PpiB was tested by molecular docking analysis. **b** The docked complex of cyclosporine-A and PpiB. The protein (pink) is shown in surface view whereas interacting residues (grey) and ligand (green) is represented in stick model. Hydrogen bond (yellow) is shown in dotted lines. **d** Interactions of PpiB with acarbose showing various hydrogen and hydrophobic interactions. **f** The docked complex of dimer of atomic gallium and PpiB. The protein (pink) is shown in surface view whereas interacting residues (green) and ligand (red) is represented in stick model. Hydrogen bond (black) is shown in dotted lines. **c**, **e**, **g** SPR analysis was performed as described in methods. Response units (RU) of the interaction of PpiB with cyclosporine-A (**c**) or acrabose **e**, or GaNP **g** from representative experiment are shown

A comparison of amino acid sequences using BLAST showed that *M.tb* PpiB exhibits at least 30% similarity with PpiB homologues in biofilm causing bacteria (supplementary Fig. S1). Results in Fig. 2a show the multiple sequence alignment of amino acid groups in the docking site of PpiB in biofilm forming bacteria that have remained conserved and could putatively interact with acarbose, cyclosporine-A or dimeric atomic gallium.^17^ Pro162 and Arg184 in *M.tb* PpiB are conserved for acarbose and cyclosporine-A binding, respectively and also present in other biofilm forming bacteria. Similarly for binding of dimer of atomic gallium,^17^ Gly203 in *M.tb* PpiB is conserved in all pathogens and the adjacent Thr204, present in the binding groove, is conserved in most of the pathogens. Homologues of *M.tb* PpiB are also present in several well known pathogenic bacteria such as *Staphylococcus aureus, Staphylococcus epidermidis, Staphylococcus intermedius, Streptococcus mutans, Staphylococcus saprophyticus, Streptococcus constellatus, Pseudomonas aeruginosa* that are known to make biofilm. It was therefore, investigated whether the amino acid residues present in the active site of PpiB, involved in interaction with acarbose or cyclosporine-A and dimeric atomic gallium, are common to other PpiB homologues in biofilm forming bacteria. The modelled structure of PpiB was found to have overall 98% residues in the allowed regions. Our model scored −1.23 in the MolProbity Clashscore was greater than the recommended Global *Z*-score values of −3, suggestive of being an adequate model. High throughput virtual screeing (HTVS), as described in methods, was done to study the probable interaction of modelled PpiB sturucture with US FDA approved drugs. These drugs were ranked in order of their docking score with PpiB (supplementary Table 1). Acarbose, with the highest docking score (−13.3), was selected for inhibition studies. Cyclosporine-A, despite having a lesser score (−5.2), was also selected as putative drug in view of its known function as cyclophilin inhibitor.^18^ Prokaryotic cyclophilins bind with cyclosporine-A with weak affinity,^14^ although not much is known about PpiB cyclosporine-A interaction.^19^ The binding site in PpiB used for docking analysis of cyclosporine-A was taken from the homologous structure of PpiB, which showed conserved docking site residue in the catalytic site.^20^ We used the Arg184 conserved residue from the catalytic centre of *M.tb* PpiB-cyclosporine-A docked complex which generated high potential energy and therefore we assumed that this protein-drug complex may represent a real entity. Multiple interactions between *M.tb* PpiB and cyclosporine-A can be seen (Fig. 2b). Molecular docking carried out using alternate platform also confirmed high binding energies of acrabose with *M.tb* PpiB. Estimated inhibition constant (Ki) was in the range of 16–20 µM for acarbose. Figure 2d and supplementary Fig. S2 show the interaction plot of acarbose with PpiB protein. Molecular interaction studies show that Thr204 interacts with dimeric atomic gallium (Fig. 2f, supplementary Fig. S3, panel c). Similarly, Gly in PpiB homologous proteins interacts with dimeric atomic gallium in most of the biofilm forming pathogens (supplementary Table 2). While the interaction of gallium to PpiB is based on dimeric nature of bonding of gallium,^17^ it remains to be shown whether the same will be true in a preparation of nanoparticles. Reports exist where atomic/molecule level docking involving specific amino acids in the target groove have been extrapolated to aggregation/complex/nanoparticle of the same (^21,22^ and references therein). These results, based on in-silico docking and high binding capacity with PpiB, point to the possibility of acarbose, cyclosporine-A and gallium in acting as inhibitors of *M.tb* PpiB. Drug docking and molecular simulations studies of PpiB with homologues proteins present in biofilm forming bacteria, annotated as WP_061736025.1, WP_049374178.1, WP_019168288.1, WP_019320573.1, WP_048792681.1, WP_006270079.1, CRQ97127.1, were also performed with acarbose, cyclosporine A and dimeric atomic gallium. Our results (supplementary Table 2) show that amino acid residues of Pro and Arg that interact with acarbose and cyclosporine-A, respectively are largely conserved across the PpiB homologous proteins, in some cases it is present at different positions. Presence of conserved amino acids at the cyclosporine-A, acarbose and gallium binding site in PpiB homologues of several biofilm forming bacteria indicate that these have also largely remained unaltered and hence could prove to be an excellent putative target across bacterial species.

Having shown in-silico binding of cyclosporine A or acarbose to PpiB, the actual physical interaction between purified recombinant *M.tb* PpiB and cyclosporine-A or acarbose, was tested using SPR spectroscopy. SPR analyses show that cyclosporine-A (Fig. 2c), acarbose (Fig. 2e) or GaNP (Fig. 2g) interact with *M.tb* PpiB in a dose dependent manner and bind with high affinity. These results suggest that cyclosporine-A, acarbose or GaNP, by virtue of their ability to bind to PpiB, could modulate the activity of PpiB.

### *M.smegmatis* expressing *M.tb* PpiB show reduced biofilm formation in presence of cyclosporine-A or acarbose or GaNP

**Fig. 3:**
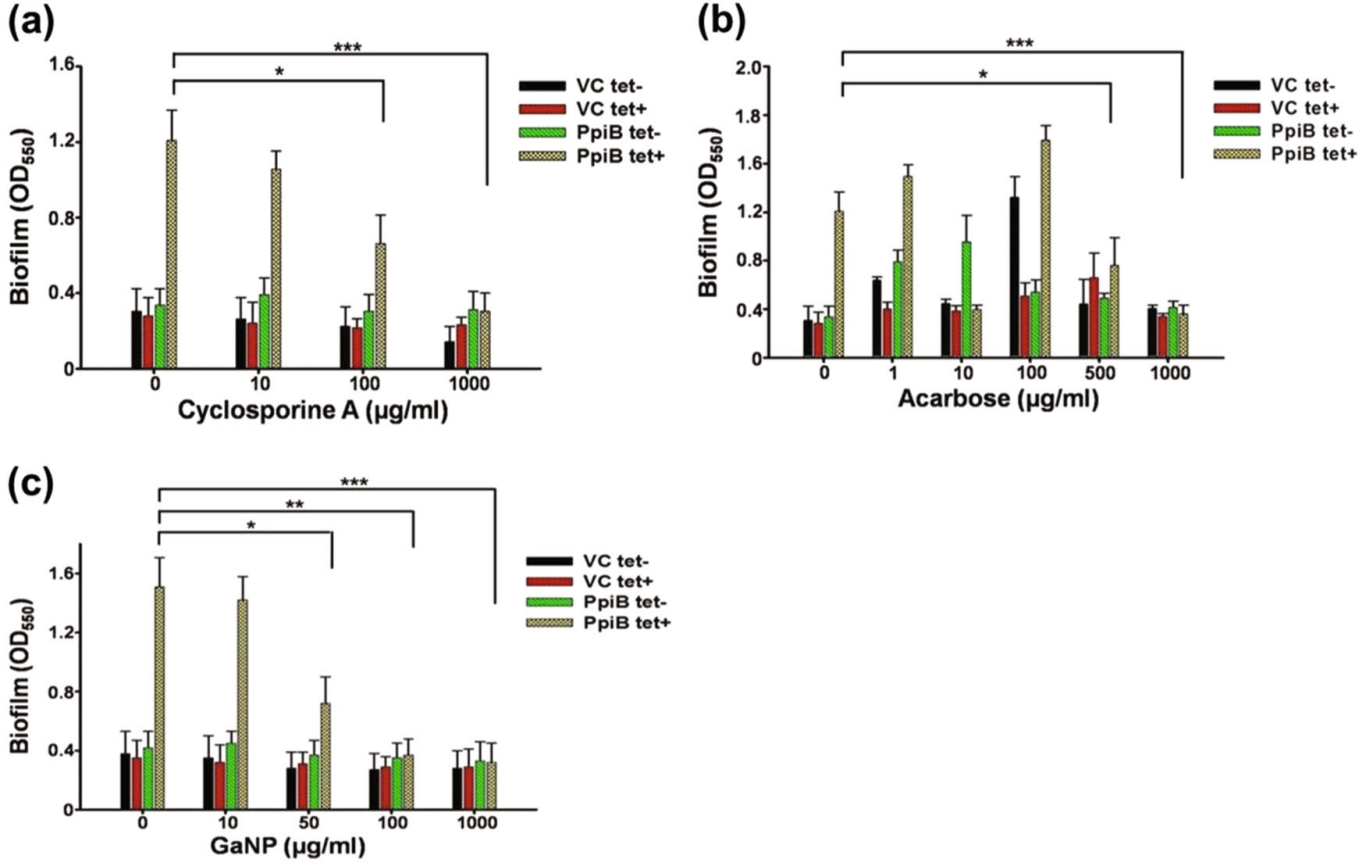
Effect of cyclosporine-A, acarbose or GaNP on induction of biofilm in *M. smegmatis* (Crystal violet assay). Ms_VC and Ms_PpiB cells were cultured in absence [()VC tet-, () PpiB tet-] or presence [() VC tet+, () PpiB tet+] of anhydrotetracycline, as described in methods. Cells were treated with cyclosporine-A (0, 10, 100, 1000 μg/ml) **a** or acarbose (0, 1, 10, 100, 500, 1000 μg/ml) **b** or GaNP (0, 10, 50, 100, 1000 μg/ml) **c** and incubated for 7 days. At the end point, biofilm was quantified as described in methods. Values shown from a representative experiment are means [±s.e.m] of biofilm formed.*p < 0.05, **p < 0.01, ***p < 0.005 (Student’s *t* test)

Given the earlier observation (Fig. 1) that *M.tb* PpiB activity is essential for biofilm formation, we speculated that modulation of PpiB activity upon binding with cyclosporine-A, acarbose or GaNP could affect biofilm formation. A threshold concentration of 100, 1000, 50 µg/ml of cyclosporine-A, acarbose and GaNP, respectively at which viability of PpiB expressing *M.smegmatis* was not significantly affected (supplementary Fig. S4) and did not showed bactericidal effect (supplementary Fig. S5) was validated using alamar blue assay. A decrease in biofilm formation may be a result of decreased cell number per-se, so it was important to ascertain the dose of cyclosporine-A, acarbose and GaNP that does not affect the overall growth of *M.smegmatis*. We accordingly assessed their effect on biofilm formation by *M.smegmatis* expressing PpiB, as described in methods. It is evident (Fig. 3) that in the absence of anhydrotetracycline induction, Ms_PpiB cells (PpiB tet-) do not develop significant levels of biofilm. Upon induction with anhydrotetracycline, Ms_PpiB cells (PpiB tet+) developed biofilm. It is apparent (Fig. 3a) that 100 µg/ml of cyclosporine-A resulted in significant decrease (*p* < 0.05) in biofilm formation while complete inhibition of biofilm formation (*p* < 0.005) was evident at a concentration of 1000 µg/ml, and this was comparable to basal levels of biofilm formation in Ms_VC cells (VC tet- or VC tet+). As shown in Fig. 3b there was a significant inhibition in the biofilm formation in the presence of 500 and 1000 µg/ml of acarbose in Ms_PpiB cells, compared to no acarbose. However, at lower concentrations of acarbose treatment, a transient increase in biofilm formation, attributed due to “Hormetic effect”, was noted in VC tet- and PpiB tet- cells.^23–25^ Significant reduction in biofilm formation compared to control (no treatment) was observed in presence of GaNP at 50 µg/ml (Fig. 3c) (*p* < 0.05). As expected, in the absence of *ppib* gene in Ms_VC cells, only basal levels of biofilm formation occurred in either presence or absence of cyclosporine-A, acarbose or GaNP. However, it was intriguing to observe that the levels of biofilm formation in Ms_PpiB cells, uninduced by anhydrotetracycline (PpiB tet-), did not exhibit any significant change to either cyclosporine-A, acarbose or GaNP administration. In Ms_PpiB cells, induction with anhydrotetracycline (PpiB tet+) showed significant decrease in biofilm formation at 100, 1000, and 50 µg/ml of cyclosporine-A, acarbose or GaNP, respectively. These in vitro biofilm inhibition results lend support to the earlier in-silico SPR results, thereby demonstrating that cyclosporine-A, acarbose or GaNP physically interact with *M.tb* PpiB in a dose dependent manner and suppress the activity of PpiB protein resulting in inhibition of biofilm formation.

### Cyclosporine-A or acarbose or GaNP co-treatment with anti-TB drugs increases susceptibility of mycobacteria to these drugs

**Fig. 4:**
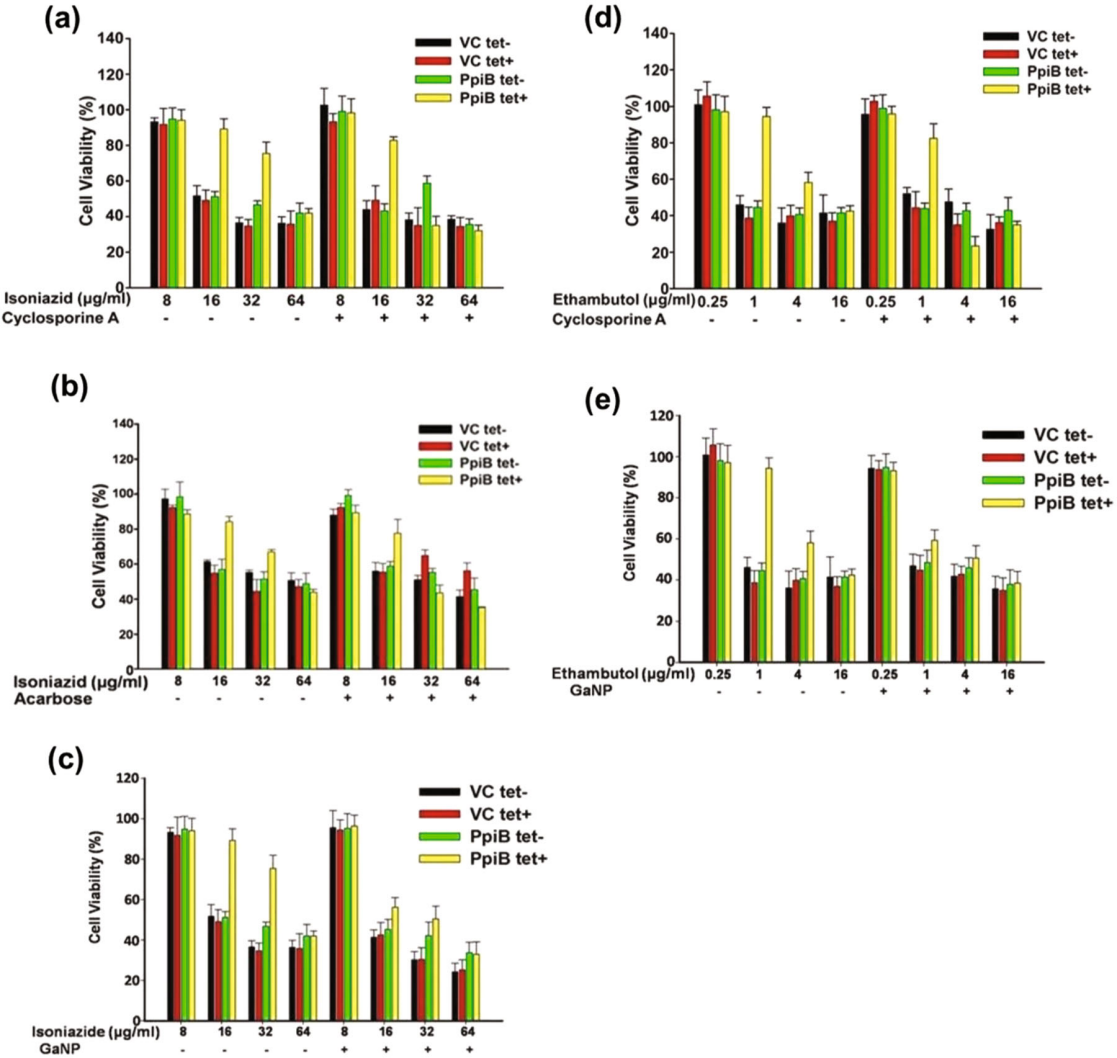
Effect of anti-TB drugs on the survival of *M. smegmatis* in the presence and absence of cyclosporine-A or acarbose or GaNP. Ms_VC and Ms_PpiB cells were induced with anhydrotetracycline to express *ppiase* in absence and presence of cyclosporine-A (100 μg/ml) **a**, **d** or acarbose (1000 μg/ml) **b** or GaNP (50 μg/ml) **c**, **e**. Cells were incubated in static culture to allow biofilm formation. At the end of 7 days Ms*_*VC and Ms_PpiB, cultured in absence of anhydrotetracycline [()VC tet-, () PpiB tet-] or presence of anhydrotetracycline [() VC tet+, () PpiB tet+], were treated either with isoniazid (0, 8, 16, 32, 64 μg/ml) or ethambutol (0, 0.25, 1, 4, 16 μg/ml) and further incubated for 68 h. Susceptibility of *M. smegmatis* to isoniazid in absence and presence of biofilm was scored by assessing the viability of cells in a 4 h alamar blue assay. Values shown from a representative experiment are means [±s.e.m] of percent cell viability

We next investigated the impact of reduced biofilm formation in terms of susceptibility to anti-TB drugs. Isoniazid and ethambutol are front line antibiotics that are normally effective at a dosage of 16 µg/ml and 1 µg/ml, respectively. Since *M.tb* PpiB expression results in enhanced biofilm formation, it could abrogate drug sensitivity of *M.smegmatis* and hence the MIC of drugs is altered. In the absence of cyclosporine-A or acarbose or GaNP, PpiB tet+ cultures, induced to form biofilm, develop physical barrier over cells and prevent exposure to anti-TB drugs. PpiB tet- cultures, that were not induced to express PpiB proteins, are unaffected by the inhibitory action of cyclosporine-A or acarbose of GaNP on biofilms and hence are exposed directly to anti-TB drugs. Results in Fig. 4a show that in the presence of cyclosporine-A (100 µg/ml), dosage of isoniazid was reduced from 64 to 32 μg/ml. Similarly, the dosage of isoniazid in the presence of acarbose (500 µg/ml), was reduced (Fig. 4b) from 64 to 32 μg/ml for PpiB (tet+). Likewise, dosage of isoniazid in presence of GaNP (50 µg/ml), was 16 µg/ml (Fig. 4c), a four-fold decrease as compared to control.

Similar experiments were carried out for ethambutol. Results (Fig. 4d) show that in the absence of cyclosporine-A, PpiB tet+ cultures exhibited dosage of 16 μg/ml for ethambutol but this decreased four-fold (4 μg/ml, *p* < 0.05) in the presence of cyclosporine-A (100 µg/ml). Results (Fig. 4e) show that dosage of ethambutol in the presence of GaNP (50 µg/ml), decreased from 16 to 1 µg/ml, as compared to control. The efficacy of acarbose in decreasing the dosage of ethambutol was insignificant (data not shown). These results clearly demonstrate that cyclosporine-A (100 μg/ml) or GaNP (50 µg/ml) inhibit the activity of PpiB protein which in turn negatively impacts the ability of the bacterium to form biofilm efficiently, resulting in reduced percent viability thereby enabling greater access of anti-TB drugs to cells. The consequent reduced viability of *Mycobacterium* in the presence of cyclosporine-A, acarbose, or GaNP points to their potential use as adjunct therapy.

### Cyclosporine-A or GaNP inhibit biofilm formation in *M.tb*

**Fig. 5:**
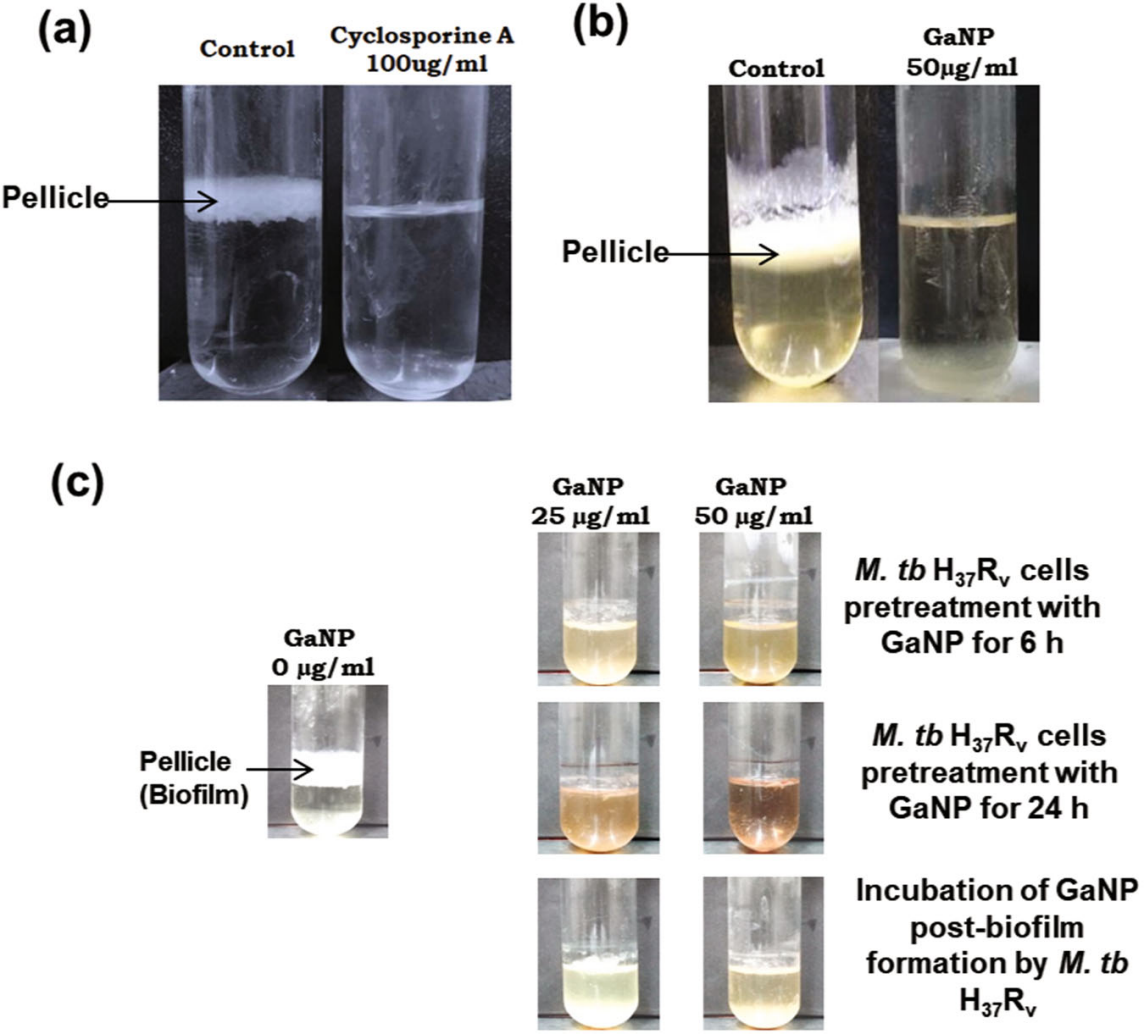
Effect of cyclosporine-A or GaNP on biofilm forming potential of *M.tuberculosis* H_37_Rv cells: H_37_Rv cells expressing PpiB were grown in static culture in absence or presence of cyclosporine-A (100 µg/ml) or GaNP (25, 50 µg/ml) for 7 days, as described in materials and methods. Representative figure of the pellicle formed at the liquid-air interface is shown. Suppressive effect of cyclosporine-A **a** and GaNP **b** on biofilm formation in *M.tuberculosis* H_37_Rv strains is evident. **c** Pretreatment or posttreatment of H_37_Rv cells with GaNP and the resultant suppression in biofilm formation

While the experiments described so far were carried out on a non-pathogenic strain of *M.smegmatis*, the eventual objective of our study was to examine if PPIase is involved in biofilm formation in virulent *M.tb* strains. Static culture of H_37_Rv cells were incubated in absence/presence of cyclosporine-A or GaNP in a BSL-3 containment facility. Results show that while untreated control H_37_Rv cells formed pellicle, treatment with cyclosporine-A (100 µg/ml) resulted in significant reduction (Fig. 5a) in biofilm formation, reduction was more pronounced in the presence of 50 µg/ml GaNP (Fig. 5b). The role of GaNP in suppressing biofilm formation was further examined in two clinically relevant scenarios. H_37_Rv cells, pretreated for 6 and 24 h with GaNP (25, 50 µg/ml), were incubated to allow biofilm formation. In another set of experiments, H_37_Rv cells were allowed to form biofilm and GaNP (25, 50 µg/ml) treatment was carried out post-biofilm formation. Pretreatment of H_37_Rv cells with GaNP (Fig. 5c) resulted in dose dependent inhibition of biofilm formation that correlated with the duration for which the cells were pre-treated, the suppression in biofilm formation being enhanced when H_37_Rv cells were pre-treated with GaNP for 24 h as compared to 6 h. Treatment with GaNP post-biofilm formation resulted in disintegration of pellicle at the liquid-air interface.

## Discussion

The current study, directed to address the problem of biofilms using Mycobacterium as model organism, explores proteins that may aid in biofilm formation and their putative inhibitors from the databank of US FDA approved drugs. Biofilm formation involves a complex process that exhibits heterogeneity in terms of the key pathways or mechanisms among different groups of microorganisms. While factors such as PrfA and SinR regulate biofilm formation in *Listeria* and *Bacillus subtilis* respectively, several other factors such as RNA regulatory proteins RsmA in *P. aeruginosa*, chaperonic proteins GroEL-1 in *M.smegmatis*, cell wall proteins PE11 in *M.tb* have also been shown to play key role in biofilm formation.^26^ There is little consensus on a single protein or factor that may act as a master molecule for biofilm formation. We therefore, started to identify unique protein that could act as putative candidate affecting biofilm formation across species. Except in *Mycoplasma genitilium* and some members of archaea, all microorganisms possess a highly conserved and ubiquitously expressed group of proteins known as cyclophilins. Cyclophilins, such as peptidyl-prolyl isomerases (PPIases; EC 5.2.1.8), catalyse the *cis*/*trans* isomerization of peptidyl-prolyl bonds and are therefore important for correct folding or refolding of nascent proteins that in turn regulate interacting partner proteins to form complexes.^27^ The role of several PPIases in biofilm formation, stress tolerance and pathogenesis of bacteria are already known.^28–30^

*M.tb* possesses two types of cyclophilins, PpiA and PpiB, of these only PpiB is essential for the survival of the pathogen as knockout variants fail to survive.^31^ Previous studies^9^ showed that *M.tb* PpiB possess chaperonic activity and aid in intracellular survival of *M.tb*. *M.tb* PpiA or PpiB genes under the control of anhydrotetracycline inducible promoter were cloned in *M.smegmatis*. Our results (Fig. 1) demonstrated that *M.smegmatis* overexpressing *M.tb* PpiB, not PpiA, developed significantly greater biomass of pellicle as compared to a basal expression in control cells. It is interesting to mention that *M.smegmatis* PpiB displays 64% homology with *M.tb* PpiB (supplementary Fig. S6). A distinct increase in biofilm formation, when compared with vector control, was expectedly seen in *M.smegmatis* over expressing *M.tb* PpiB. Glycopeptidolipids, like PpiB, are component of the membrane fraction and are also part of the secretome and are known to play important role in biofilm formation.^32–34^ Our results clearly demonstrate a direct involvement of *M.tb* PpiB in biofilm formation. That PpiB also acts as a chaperone^9^ is in agreement with reports of staphylococcus trigger factor having roles in stress tolerance and biofilm formation.^32^

Our next step was to identify suitable drug(s) that could act as an inhibitor of PpiB protein. Developing new drugs is a long process taking about 10–15 years. Drug repurposing is gaining popularity as it allows bypassing of the cumbersome clinical trial of drugs for which the parameters of toxicity and effectiveness have already been tested and approved. The effectiveness of osteoarthritis drug Celebrex in decreasing polyp formation in colon cancer patients, anti-malarial drug chloroquine in improving outcome of cancer drug Erlotinib, anti-diabetic drug metformin in lowering morbidity of TB patients are some examples of drug.^35^ An inhibitor of human phosphodiesterase, sildenafil originally used in case of erectile dysfunction, has shown encouraging results in animal studies and is now being deliberated as an adjuvant host directed therapy to curtail the duration of TB drug regimen.^36^ Nanoparticles are also emerging as key modulators against several human pathogens. FDA approved gallium has shown promising efficacy against *M.tb* due to similar charge as Fe, thereby allosterically competing with Fe to bind Fe-siderophores.^37^ This results in disruption of iron metabolism, leading to failure of microbial cells to grow in presence of gallium.^38^

Cyclophilin inhibitors, as a unique tool in therapeutic biology, are showing promising results in several diseases.^39^ Previous studies^19^ pointed that PpiA is a Cyclosporine-A binding cyclophilin and treatment with cyclosporine-A sensitises drug-tolerant biofilm of *Candida albicans* to various antifungal drugs.^40,41^ We used *M.smegmatis* over expressing *M.tb* PpiB as a model to evaluate the effect of known and unknown inhibitors of cyclophilins. In-silico docking analysis of interaction of cyclosporine-A with *M.tb* PpiB homologue revealed that PipB possesses conserved amino acid groups in the binding pocket. These molecular docking studies, in sync with previous studies, show that Cyclosporine-A can stereochemically bind with PpiB. Among the FDA approved drugs, acarbose exhibited greatest docking score and could potentially interact with PpiB. The physical interaction of cyclosporine-A, acarbose and GaNP with PpiB was experimentally confirmed through SPR studies. Consistent with these results, we showed that cyclosporine-A, acarbose or GaNP could suppress biofilm formation. A biphasic dose response for biofilms has been reported for many inhibitors/antibiotics/chemicals/drugs/ligands etc. Such a response, termed as “Hormetic response”, is characterised by stimulation of biofilm formation at lower dose and inhibition of the same at higher dose. Some reports have also pointed out to antibiotic acting as antagonist of biofilm formation at low levels, agonists at higher levels and once again antagonist at still higher level.^23–25^ This is exactly what we have observed: acarbose at lower concentration (upto 100 µg/ml) showed increase in biofilm formation and at 500 µg/ml and above inhibited biofilm formation. While cyclosporine A and acarbose exhibit bacteriostatic activity at the concentration reported (supplementary Fig. S5), only at higher concentration GaNP exhibits bactericidal effect.

A comparison of the amino acid domains in the binding pocket of PpiB homologues expressed in biofilm forming microorganisms interestingly showed that PpiB possess similar amino acids that can interact with either cyclosporine-A or acarbose or GaNP. The structure of gallium nanoparticle has not been reported so far, we therefore used dimeric form of atomic gallium^17^ that could act as the building block of gallium nanoparticle. This clearly positions PpiB as a unique protein that can be targeted to inhibit biofilm formation across bacterial species, more so when several mixed species of microorganisms exist in the biofilm. Each of these heterogenous species develop biofilms using varying cellular pathways. Although some antibiotics act as anti-biofilm agents, however, such a drug that may be effective against a putative protein involved in biofilm formation may not be as effective in other organisms either due to the absence of the protein target or redundancy in the metabolic pathway. The presence of conserved amino acid Arg, Pro, Gly at the binding site of Cyclosporine-A, acarbose and GaNP, respectively highlights that PpiB could prove to be a unique target in controlling biofilms, thereby providing a possible generic mechanism for treatment of infections caused by other biofilm producing pathogens.

**Fig. 6:**
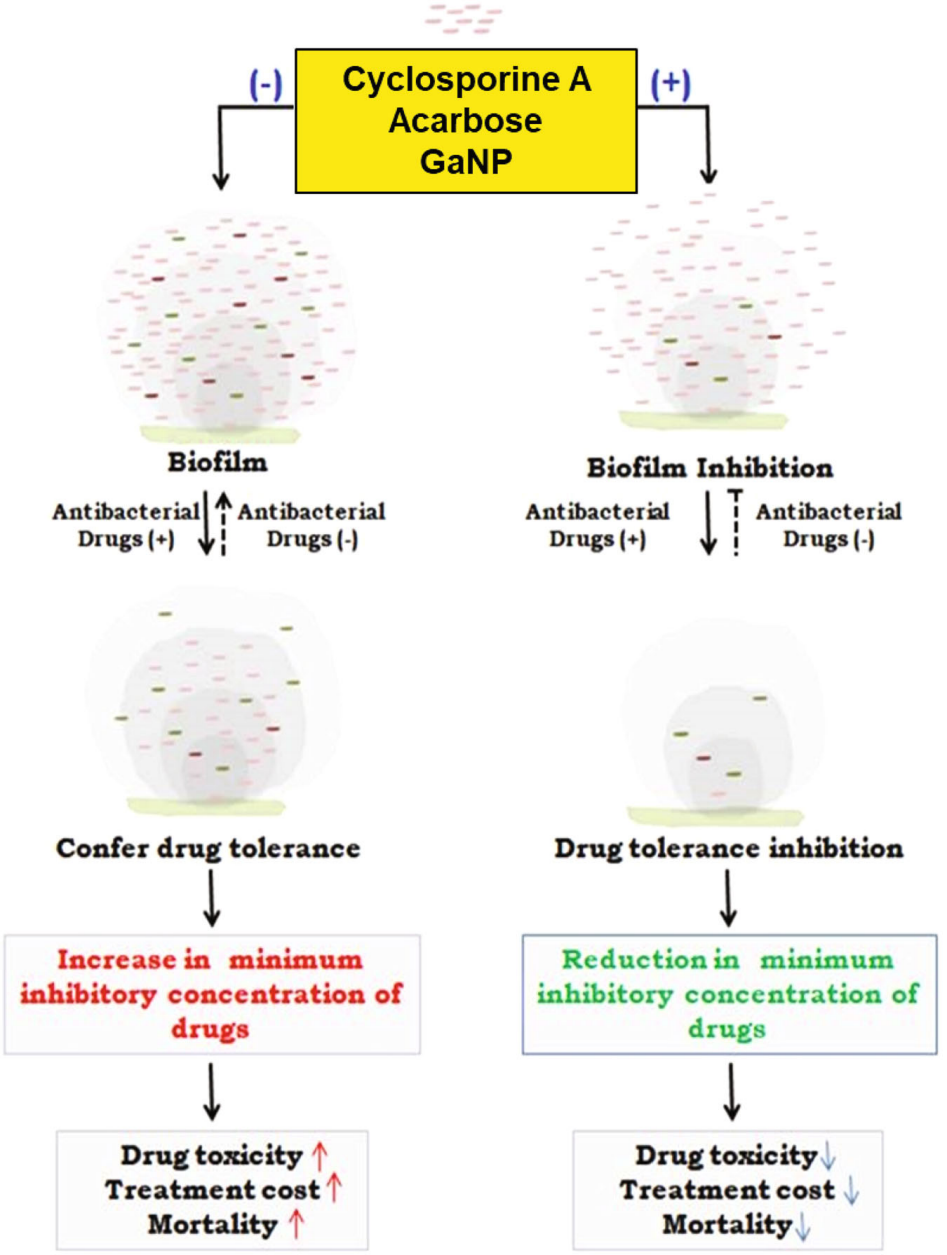
Schematic overview of the effect of repurposed drugs on biofilm and its outcome on tuberculosis treatment: Under stress like conditions Mycobacteria secrete exogenous layer of matrix that forms a physical barrier for entry of drugs. The cells within the matrix continuously secrete to develop a biomass of biofilm that enables the cells to withstand high minimum inhibitory concentration (MIC) of drugs. As a result, higher dosage of drugs is required to kill the cells. Cells at the core of the biofilm matrix are least affected by drugs and evolve in due time so as to withstand even higher concentration of drugs. This confers drug tolerance and leads to drug toxicity, increased treatment cost and mortality. Cyclosporine-A, acarbose and GaNP inhibit the activity of PpiB that play crucial role in biofilm formation. Treatment with these drugs suppresses formation of biofilm and the bacterium is exposed directly to the drugs. As a result the drug is effective at low MIC values. Treatment with these drugs also reduces the MIC of existing anti-tubercular drugs resulting in decreased toxicity. The end result is that patient mortality and treatment cost may be reduced significantly. Regular and dotted arrows in the figure denote confirmed and putative roles respectively

While there are global efforts to develop new drugs against TB, efforts are needed to reduce the duration of the drug regimen. Using first line anti-TB drugs (ethambutol and isoniazid), we have shown that the reduced mycobacterial biofilm formation in the presence of cyclosporine-A, acarbose or GaNP (Fig. 3) results in dosage reductions for these anti-TB drugs (Fig. 4). While increased dosage of anti-TB drugs results in drug tolerance of the pathogen, it also has a negative impact on patients in terms of toxicity. Our results show that treatment with cyclosporine-A or acarbose help in reducing the dosage of anti-TB drugs by at least two-fold. This has wide implications as it provides proof of principle that cyclosporine-A, a known immunosuppressant that affects T cells, can be repurposed as a conjunct therapy against biofilm associated diseases (Fig. 6). One can argue that treatment with cyclosporine-A may activate latent TB by suppressing immunity. It is conceivable that the concentration of cyclosporine-A at which it inhibits biofilm formation can be reduced further to minimal concentrations by using this drug with suitable adjuvants, thereby reducing chances of its immunosuppressive effects to prevail over its efficacy as biofilm inhibitor. Our results related to the use and efficacy of cyclosporine-A is in line with previous reports that suggest that it acts synergistically to improve the efficacy of antifungals against *C.parapsilosis*.^42^ Cyclosporine-A in combination with azole antifungal flucanizole has been shown to be effective against biofilms formed by *C.albicans*^43^ and also imparts sensitivity to *C.albicans* towards fluconazole by involving multiple pathways.^44^ GaNP is known to facilitate phagosome maturation, inhibit growth of *M.tb* in macrophages, inhibit HIV infection through release of interferons and can be targeted to human macrophages infected with both *M.tb* and HIV.^45,46^ These studies support our results and point to the possibility that GaNP would be an effective intervention against bacterial biofilms. It has not escaped our attention that cyclosporine-A as an adjunct to existing anti-tubercular drugs could be a potential strategy to address the problem of eradicating latent TB by first activating the bacterium, by virtue of its immune-suppressive action, followed by the biofilm inhibition reported in our study.

We also show that acarbose treatment resulted in a reduction of dosage of anti-TB drugs such as isoniazid. Acarbose is widely used for the management of type 2 Diabetes mellitus as well. The efficacy of acarbose to block maltose importer and consequently suppress growth of *E. coli* is known.^47^ Given the fact that TB and diabetes exhibit distinct correlation in patients and synergistically affect the clinical outcome of each other in patients, the efficacy of acarbose as a medicament in reducing the dosage of anti-TB drugs could prove to be beneficial. There are several bacteria that are involved in the biofilm of cystic fibrosis, wounds, contact lenses, orthopaedic implants, breast implants, dental biofilm, pacemakers, prosthetic heart valves etc. and have similar protein to *M.tb* PpiB.^48^ Our results can be extrapolated to test the efficacy of cyclosporine-A or acarbose or GaNP in reducing the dosage of other drugs or for diseases caused by biofilm-forming microorganisms as well.

Taken together, these results conclusively demonstrate that PpiB is a potential drug target involved in Mycobacterium biofilm formation and cyclosporine-A, acarbose and GaNP directly bind to PpiB and disrupt biofilm formation. The consequent reduction in dosage values for anti-TB drugs ethambutol and isoniazid points to their ability to act as therapeutic interventions to counter drug tolerance and also possibly reduce dosage of existing anti-tubercular drugs with implications in reducing drug-induced toxicity and also treatment duration. It will also be interesting to evaluate PpiB as a drug target given the fact that PpiB is not only an essential gene of *M.tb*, but is involved in biofilm formation. The conservation of drug binding sites within PpiB across pathogenic bacteria biofilm tempts us to suggest that PpiB-targeted biofilm disruption could prove to be a masterstroke for combatting biofilm-related infections across microbial species.

## Methods

### Reagents

*M.smegmatis* mc^2^155, initially obtained from ATCC, was maintained in our laboratory as glycerol stocks. *M.tb* H_37_Rv (gift from Prof R. K. Bhatnagar, Jawaharlal Nehru University, New Delhi, India), was cultured in BSL-3 facility. Growth media (Middlebrook 7H9) and OADC supplement were obtained from BD, USA. Glycerol, Tween 80, acetic acid, acarbose and cyclosporine-A were procured from Sigma-Aldrich, India. GaNP (purity > 99.9%) was obtained from Nanoshel, India. All other reagents such as Alamar Blue, Crystal Violet, Isoniazid, and Ethambutol were of analytical grade and obtained from Himedia, India.

### Constructs and recombinant strains used in the study

Recombinant strains of *M.smegmatis* expressing *M.tb* Ppiases were generated using *E. coli*-mycobacterium shuttle vector pST2K and specific oligonucleotide primers, as detailed elsewhere.^10^ Briefly, pST_ppiA, pST_ppiB and pST2K vector containing anhydrotetracycline inducibe promoter were electroporated in wild type *M.smegmatis* (Ms_WT) and transformed strains were designated as Ms_PpiA, Ms_PpiB, and Ms_VC, respectively.

### Cell culture and biofilm formation

*M.smegmatis* and *M.tb* H_37_Rv were maintained in growth media supplemented with 10% OADC, 0.001% glycerol, and 0.05% Tween-80. Cultures were incubated at 37°C in a shaker incubator and diluted to OD of 0.08 in growth media prior to sub-culturing in 96-wells or in test tubes for induction of biofilms. 20 ng/ml anhydrotetracycline was used to induce PPIase expression in recombinant *M.smegmatis*, resulting in biofilm formation. Recombinant *M.smegmatis* and *M.tb* H_37_Rv were cultured in static phase in Tween 80-free growth media for 7 days and 4 weeks, respectively to allow formation of pellicle at the liquid air interface.

### Crystal violet assay

Cyclosporine-A, acarbose, and GaNP effect on biofilm formation was assessed by quantifying the pellicle formed at the liquid air interface, using crystal violet.^49^ Anhydrotetracycline induced Ms_PpiB and Ms_VC cells were cultured in the presence of various concentrations of cyclosporine-A (0, 10, 100, and 1000 µg/ml), acarbose (0, 1, 10, 100, 500, 1000 µg/ml) or GaNP (0, 10, 50, 100, 1000 µg/ml) in sterile flat bottom 96-well microtiter plate (Thermo Scientific, India). At the end of 7 days of static phase culture, the media beneath the pellicle were aspirated out and remaining solid pellicle was stained by adding 125 µl (w/v) 0.1% Crystal Violet solution. The stained pellicle was washed thrice with water followed by addition of 30% acetic acid. The samples were subsequently incubated for 10–15 min at room temperature to dissolve the stain and absorbance was spectrophotometrically recorded at 550 nm.

### Percent viability of biofilm induced culture

Biofilm formation by *M.smegmatis* Ms_VC and Ms_PpiB induced with or without anhydrotetracycline was performed in presence of FDA approved agents, as described above. At the end of 7 days of incubation period isoniazid (8, 16, 32, 64 µg/ml) or ethambutol (0.25, 1, 4, 16 µg/ml) was added to the wells of the microtiter plate and the plate was incubated further for 68 h. Cell viability in presence of isoniazid and ethambutol were assessed using Alamar Blue assay.^50^ Briefly, 0.01% alamar blue reagent was added to each well of microtiter plate and the plates were further incubated for 3–4 h. Conversion of resazurin (blue) to resorufin (pink) was monitored at 570 nm and 600 nm, respectively to score the viability of the cells. All assays were performed in triplicate.

### In-silico amino acid sequence alignment and similarity search

The amino acids sequences of *M.tb* PpiA (GenBank accession number: CCP42731.1), *M.tb* PpiB (GenBank accession number: CCE38048.1 were downloaded from the *NCBI*. The sequence homology search of *M.tb* PpiB was done using BLASTp in known biofilm forming bacteria on NCBI website*. M.tb* PpiB amino acids sequence was used as queries in BLASTp analyses against the NCBI non-redundant protein database of the specific bacteria to find their similar homologues.

### Modelling of PpiB structure and molecular dynamics (MD) simulations

Crystallographic structure of PpiB, being unavailable at Protein Data Bank, homology modelling techniques involving multiple bioinformatics tools or servers such as the MODELLER version 9.11 or Phyre2, respectively were used to generate PpiB model structure. Protein sequence of PpiB from *M.tb* (strain ATCC 25618/H_37_Rv) was obtained from UniprotKB database [P9WHW1]. Protein structure model validation was carried out using protein structure validation software suite (PSVS).

### Molecular docking analysis of *M.tb* cyclophilin (PpiB) in complex with cyclosporine-A, acarbose, or gallium

Molecular docking analysis of cyclosporine-A was carried out to study the interactions and affinity with the PpiB protein using AutoDock Tools 1.5.6 (open access). 3D structure of cyclosporine-A was obtained from chemical structure database ChemSpider. AutoDockVina 1.1.2 program was used for docking of cyclosporine-A at the docking site of PpiB protein. The location of the catalytic site was mapped and deduced from the structure-based alignment of related proteins reported earlier.^20^

Alternatively, Glide module of Schrodinger was used to screen other compounds from FDA library as described previously.^51^ Briefly, Drug library was prepared using Ligprep module applying OPLS 2005 force field and docking was performed using HTVS and XP (extra precision) docking to filter out the compounds with low binding energy. Compounds having a docking score greater than −5 in HTVS were used for XP docking protocol. An XP score greater than −8 was scored as strong binding. The dynamic nature of interaction between PpiB and acarbose was studied using GROMACS version 4.6.5 and above assigning GROMOS96 43a1 force field as per standard protocols.

The chemical structure of elemental gallium was obtained from PubChem (CID 5464084). Using the elemental gallium, a dimer structure for atomic gallium was created using Maestro interface available from Schrodinger. Molecular orbital analysis of gallium in dimeric state showed that gallium dimer is the essential building block for the formation of gallium clusters.^17^ Further molecular docking study of dimeric atomic gallium was carried out with PpiB protein and its homologues using PatchDock algorithm.^52^ Ligplot was used for visualisation of the interactions between protein-ligand complex in 2D schematic representations. PyMol and Chimera were used for preparing cartoon representations of the structures.

### Surface plasmon resonance (SPR) study

In-vitro interactions of *M.tb* PpiB with cyclosporine-A, acarbose and GaNP were studied using SPR technique employing Autolab ESPRIT analyzer, as per manufacturer’s instructions. Briefly, *M.tb* rPpiB^9^ (expressed in *E. coli* BL21(DE3) rosetta strain) was diluted in PBS upto a concentration of 100 μg/ml and passed through a CM5 sensor chip for immobilisation. During association phase (300 s) cyclosporine-A, acarbose and GaNP were diluted to different concentration in running buffer (PBS) and were allowed to pass over the immobilised PpiB. During dissociation phase (150 s) PBS was applied to sensor chip and the sensor chip was regenerated between each binding experiment with NaOH.

### Statistical analysis

Statistical analyses using a two-way analysis of variance (ANOVA) and Student’s *t*-test were performed using Sigmaplot and Sigmastat software (Systat Software Inc, San Jose, CA). A *p*-value (*p* < 0.05) was considered significant.

## Supplementary information

Supplementary Information

## Data Availability

The authors declare that all the data supporting the findings of this study are available within the article, or upon request from the corresponding author.

## Supplementary information

**Supplementary information** accompanies the paper on the *npj Biofilms and Microbiomes* website (10.1038/s41522-018-0075-0).
