# Supplementary material for: Peptidyl-prolyl isomerase-B is involved in Mycobacterium tuberculosis biofilm formation and a generic target for drug repurposing-based intervention
Source: NPJ Biofilms Microbiomes. 2019 Jan 15;5:3. doi: 10.1038/s41522-018-0075-0 (PMC6333787; doi:10.1038/s41522-018-0075-0)
Supplement: Supplementary file 1 — Supplementary Information [file 41522_2018_75_MOESM1_ESM.pdf]

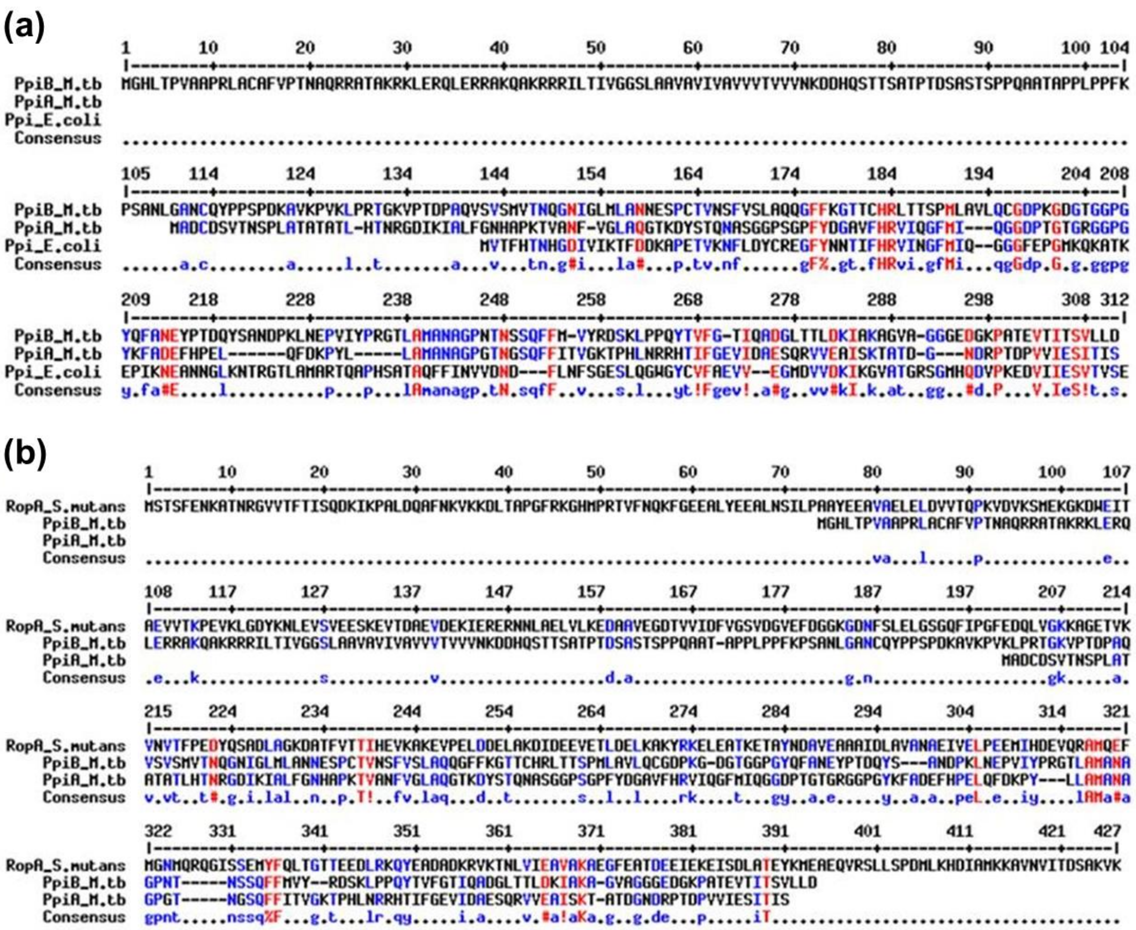

Figure S1: Sequence alignment and comparison of different PPiase. (Panel a): Multiple sequence alignments using BLASTp were performed to assess similarities between *M.tb* PpiB, *M.tb* PpiA and *E.coli* peptidyl-prolyl isomerase. (Panel b): Multiple sequence alignments of *M.tb* PpiB, *M.tb* PpiA and peptidyl-prolyl isomerase RopA (trigger factor) of *S.mutans* were tested using BLASTp. Highly conserved and less conserved amino acids are shown in red and blue, respectively.

M.tb PpiA or PpiB exhibit high degree of similarity in conserved amino acids found in *E.coli* PPIase. *M.tb* PpiB also possesses an extended sequence of 100 amino acids in the N-terminal end and is absent in either *M.tb* PpiA or *E.coli* PPIase. That *M.tb* PPIase exhibits high degree of sequence similarity with RopA proteins expressed in *S.mutans* is evident.

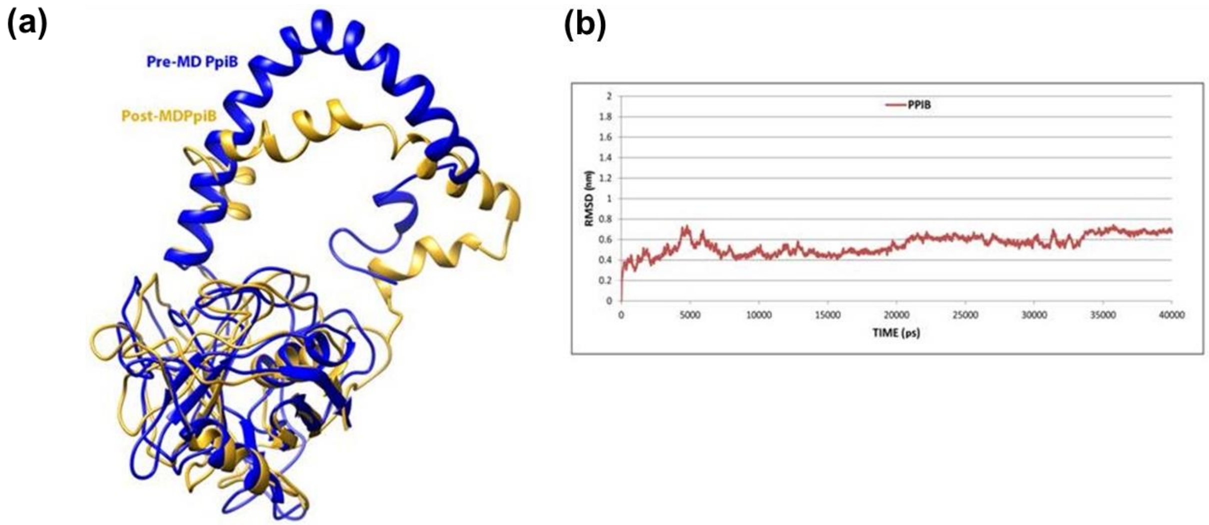

Figure S2: Molecular Modelling of PpiB and *in-silico* docking of acarbose with PpiB. The modeled structure of PpiB obtained using Phyre2 or MODELLER was found to have overall 98% residues in the allowed regions. Our model scored -1.23 in the MolProbity Clashscore that was greater than the recommended Global Z-score values of -3, suggestive of being an adequate model. Verify3D also corroborated the reasonable quality of the model. (Panel a): Ribbon representation showing superimposition of the Pre-MD PpiB (shown in blue) and Post-MD PpiB structure obtained from the 40 ns molecular dynamic simulations (shown in golden-yellow). (Panel b): RMSD plot of PpiB apo structure molecular dynamics trajectory.

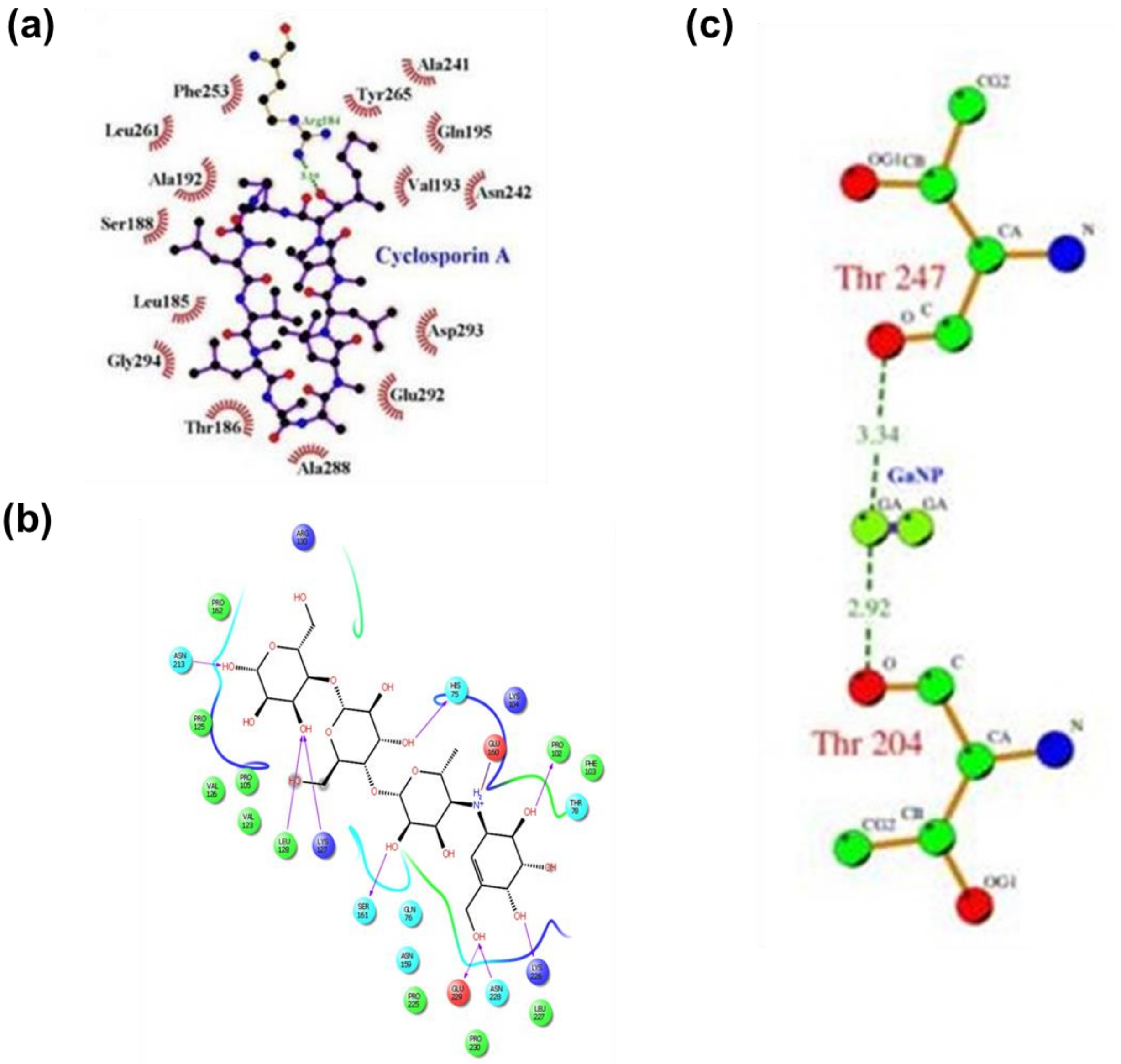

Figure S3: Ligplot of interactions of PpiB with Cyclosporine-A, acarbose and dimeric atomic gallium. (Panel a): Ligplot of interacting amino acid residues of PpiB interacting with cyclosporine-A. (Panel b): Ligplot of interacting amino acid residues of PpiB interacting with acarbose. (Panel c): Ligplot of interacting amino acid residues of PpiB interacting with dimeric atomic gallium[17].

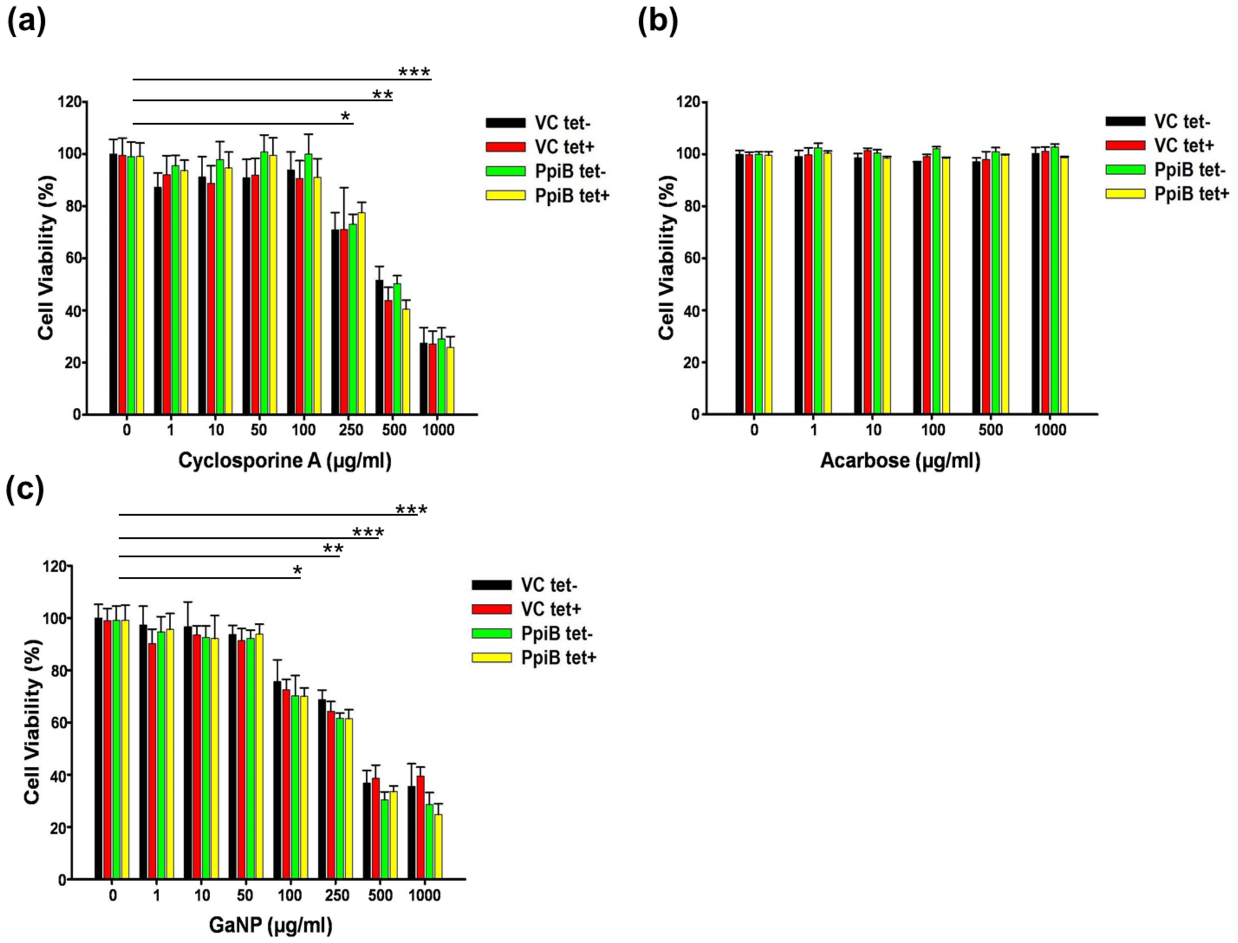

Figure S4: Cyclosporine-A, acarbose or GaNP impact the viability of *M.smegmatis*. Ms_VC and Ms_PpiB cells were induced with anhydrotetracycline to express *ppiase* gene in absence and presence of cyclosporine-A (0, 1, 10, 50, 100, 250, 500, 1000 µg/ml) (Panel a), acarbose (0, 1, 10, 100, 500, 1000 µg/ml) (Panel b) or GaNP (0, 1, 10, 50, 100, 250, 500, 1000 µg/ml) (Panel c), as described in methods. At the end of incubation period, cell viability was assessed in a 4 h alamar blue redox assay. Values shown from a representative experiment are means [±SEM] of percent cell viability for *M. smegmatis* (VC and PpiB) cultured in absence [( )VC tet-, ( ) PpiB tet-] or presence [( ) VC tet+, ( ) PpiB tet+] of anhydrotetracycline. Note the decline in cell viability evident at higher concentration of only GaNP but not cyclosporine A or acarbose. *p<0.05, **p<0.01, ***p<0.005 (ANOVA).

Initially, the effect of acarbose, cyclosporine-A and GaNP on the viability of *M. smegmatis* cells expressing *M. tb* PpiB was examined colorimetrically using Alamar Blue redox dye assay, as described in methods. Control (Ms_VC) and recombinant (Ms_PpiB) strains were induced with anhydrotetracycline, hereafter referred as VC tet+ and PpiB tet+, respectively. Ms_VC and Ms_PpiB not induced with anhydrotetracycline were used as control, hereafter referred as VC tet- and PpiB tet-, respectively. It can be seen (supplementary Fig. S4, Panel a) that cyclosporine-A below 250 µg/ml had insignificant effect on the number of viable *M. smegmatis* cells. However, a comparison of the cell viability of the group, [VC (tet-/tet+) and PpiB (tet-/tet+)], treated with 250 µg/ml and above of cyclosporine-A with the control group (no treatment of cyclosporine-A), showed significant difference ($P<0.05$, $P<0.01$ and $P<0.005$) in viable cell numbers. Acarbose up to (1000 µg/ml) had no effect on the number of viable Ms_VC or Ms_PpiB cells (supplementary Fig. S4, Panel b). Even at 10 mg/ml, acarbose showed no significant effect on cell viability (data not shown), implying that it did not exhibit inhibitory effect on survival of *M. smegmatis.* Results in supplementary Fig. S4 (Panel c) show that GaNP below 100 µg/ml had no significant effect on the number of viable *M. smegmatis* cells. Cell viability of the group, [VC (tet-/tet+) and PpiB (tet-/tet+)] treated with 100 µg/ml and above of GaNP, with the control group (no treatment of GaNP), shows that there is significant difference ($P<0.05$ $P<0.01$ and $P<0.005$) in viable cell number. A threshold value of 100 µg/ml, 1000 µg/ml and 50 µg/ml of cyclosporine-A, acarbose and GaNP, respectively was selected for further experiments to examine the effect of these components on biofilm formation. These threshold values of cyclosporine-A, acarbose and GaNP at which viability of PpiB expressing *M. smegmatis* is not affected was crucial for validating the effect of these components on biofilm formation. A decrease in biofilm formation may be a result of decreased cell numbers *per-se*, so it was important to ascertain the threshold dose of cyclosporine-A, acarbose or GaNP that does not affect the overall growth of *M. smegmatis.*

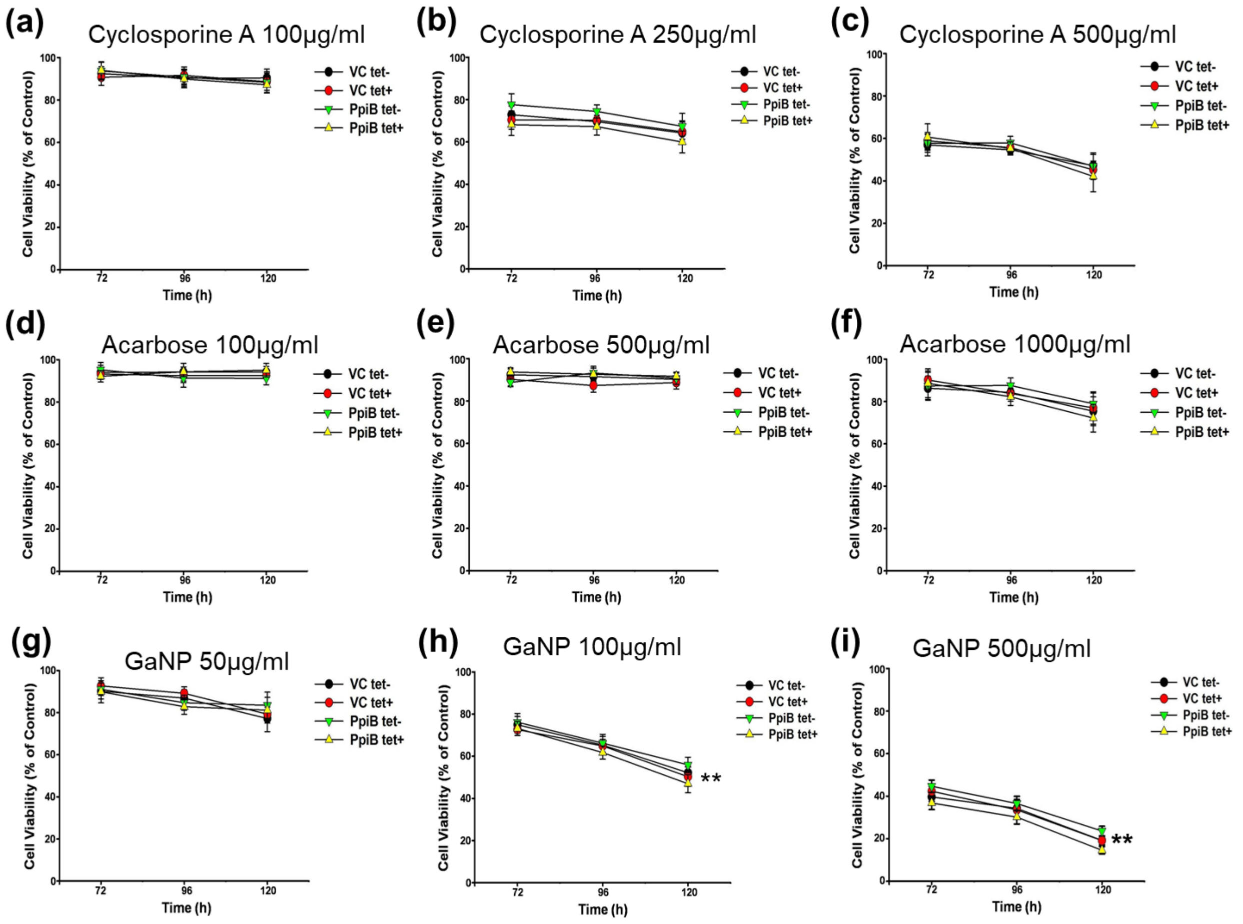

Figure S5: Bacteriostatic *versus* bactericidal effect of cyclosporine-A, acarbose and GaNP. Ms_VC and Ms_PpiB cells were induced with anhydrotetracycline to express *ppiase* gene in absence and presence of cyclosporine-A (100 µg/ml, Panel a; 250 µg/ml, Panel b; 500 µg/ml, Panel c), acarbose (100 µg/ml, Panel d; 500 µg/ml, Panel e; 1000 µg/ml, Panel f) or GaNP (50 µg/ml, Panel g; 100 µg/ml, Panel h; 500 µg/ml, Panel i), as described in methods. At the end of incubation period (72 h, 96 h, 120 h), cell viability was assessed in a 4 h alamar blue redox assay. Values shown from a representative experiment are means [±SEM] of percent cell viability for *M.smegmatis* (VC and PpiB) cultured in absence [(-●-)VC tet-, (-▼-) PpiB tet-] or presence [(-●-) VC tet+, (-△-) PpiB tet+] of anhydrotetracycline. **p<0.01(ANOVA). Note the bacteriostatic effect of cyclosporine-A, acarbose and GaNP (panels a to g). In panels c, f and g the values at time 72 h and time 120 h are not significant. However, in panels (h) and (i), a very significant decline in

growth at time 120 h is evident when compared with 72 h, pointing to the bactericidal effect of GaNP.

In order to assess whether cyclosporine-A, acarbose and GaNP has bacteriostatic or bactericidal effect on *M. smegmatis,* cells were cultured with these drugs for upto 120 h. A significant decrease in cell viability of the treatment group at 120 h as compared to at 72 h denoted bactericidal effect of the drug. Results in supplementary Fig. S5 show that at 100 μg/ml, both cyclosporine-A and acarbose exert bacteriostatic effect. It is also evident that cyclosporine-A, acarbose and GaNP have bacteriostatic effect upto 500 μg/ml, 1000 μg/ml, 50 μg/ml, respectively (supplementary Fig. S5, panels a to g). The apparent decline in the values at time 120 h as compared to at 72 h (supplementary Fig. S5, panels c, f and g) were not significant based on ANOVA analysis. A very significant decline in growth at time 120 h was evident (supplementary Fig. S5, panels h, i), pointing to bactericidal effect of GaNP at 100 μg/ml and above. These results indicate that cyclosporine-A and acarbose have bacteriostatic effect on cell viability whereas GaNP at higher concentration is bactericidal.

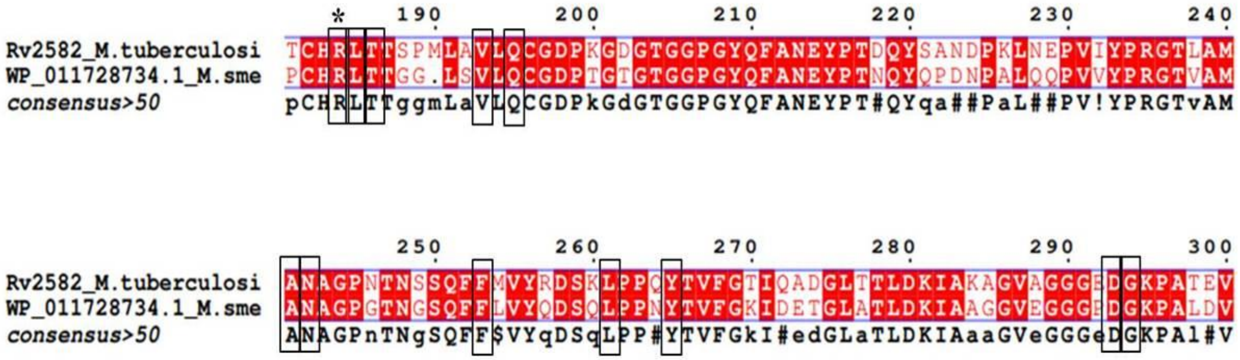

Figure S6: Sequence alignment of Rv2582 (PPIB protein) from *M. tuberculosis* and WP_011728734 from *M.smegmatis*. The binding pocket residues present in Rv2582 are Ala241, Tyr264, Gln195, Val193, Asn242, Asp293, Thr186, Gly294, Leu185 Ser188, Ala192, Leu261, Phe253, Arg184, Glu292, Ala288, Ser188, Ala192. Sequence alignment showed that all the above mention residues are also conserved in WP_011728734 from *M.smegmatis* except Glu292, Ala288, Ser188 and Ala192 and t6e conserved residue Arg184 from PPIB protein which was used as catalytic centre for docking with cyclosporine-A is also conserved in WP_011728734 gene.

Supplementary Table 1: Docking results of PpiB with FDA Approved Drugs

	GENERIC NAME	PRODUCTS	DOCKING SCORE	FUNCTIONS
1	Acarbose	Acarbose; Glucobay; Precose	-13.3	Reversible binding to pancreatic alpha-amylase & membrane-bound intestinal alpha-glucoside hydrolases. Used for treatment of diabetes type II.
2	Cyclosporine A[#]	Gengraf, Sandimmune	-5.2	It is an immunosuppressant used in patients undergoing organ transplantation. It is also used for treatment of psoriasis and severe rheumatoid arthritis.
2	Diosmin		-12.2	It is a semisynthetic drug used for the treatment of venous diseases.
3	Ouabain		-9.8	It inhibits Na-K-ATPase membrane pump and used in treatment of atrial fibrillation & heart failure.
4	Ticagrelor	Brilinta	-9.7	It blocks ADP receptors.<br>Used for prevention of thrombotic events such as stroke & heart failure.
5	Flavin adenine dinucleotide		-9.5	It is used to treat eye diseases due to vitamin B2 deficiency.
6	Travoprost	Izba; Travatan Z	-9.4	It is a selective prostanoid receptor agonist that is used to reduce elevated intraocular pressure.
7	Cytarabine	Cytarabine;Cytosar	-8.9	It acts by direct DNA damage as well as incorporation into DNA. Used in treatment of different forms of leukaemia.

8	Lymecycline		-8.7	Inhibits cell growth via inhibition of translation and used for treatment of infections & acne.
9	Azacitidine	Azacitidine; Vidaza	-8.7	Inhibits the DNA methyltransferase at low doses while incorporates into DNA and RNA at high doses, resulting in cell death.<br>Used for treatment of patients with French-American-British myelodysplastic syndrome subtypes.
10	Paromomycin		-8.5	Inhibits protein synthesis via 16S ribosomal RNA binding.<br>Used for treatment of acute as well as chronic intestinalamebiasis.
11	Adenosine monophosphate (AMP)		-8.3	Dietary supplement to boost immune activity. Also used as substitute sweetener for low-calorie diet.
12	Daunorubicin	Daunorubicin Hydrochloride; Daunoxome	-8.3	It forms complexes with DNA thereby having cytotoxic and antimitotic activity. Used in the treatment of nonlymphocyticleukaemia.
13	Epirubicin	Ellence	-8.3	Inhibits nucleic acid and protein synthesis via different mechanisms.<br>Used in adjuvant therapy for patients with axillary node tumor involvement.
14	Doxorubicin	Caelyx; Doxil; Myocet	-8.3	Intercalates between base pairs and inhibits topoisomerase II activity

				thereby having antimitotic and cytotoxic activities.<br>Used in treatment of acute myeloblasticleukemia and acute lymphoblastic leukemia,
15	Valrubicin	Valstar;<br>Valtaxin	-8.2	An anthracycline that affects nucleic acid metabolism and used in bladder cancer treatment.
16	Pentostatin	Nipent	-8.1	A transition state inhibitor of ADA(adenosine deaminase) and used in treatment of hairy cell leukaemia refractory to alpha interferon.
17	Sofosbuvir	Sovaldi	-8.1	A nucleotide analog inhibitor, that inhibits HCV NS5B polymerase and used in combination therapy for treatment of chronic hepatitis C virus.
18	Glyburide	Diabeta;<br>Euglucon;<br>Glyburide;<br>Glynase	-8.1	It binds to ATP-sensitive potassium channels on pancreatic cell surface and used as an adjunct to diet for lowering blood glucose in patients with NIDDM.
19	Fludarabine	Fludara;<br>Fludarabine;<br>Fludarabine Phosphate;<br>Oforta	-8.1	It gets converted to 2-fluoro-ara-ATP and this metabolite inhibits DNA synthesis.<br>Used for treatment of B-cell chronic lymphocytic leukaemia
20	Pioglitazone	Actos;<br>Pioglitazone;<br>Hydrochloride	-8.1	Agonist of peroxisome proliferator activated receptors (PPAR) and used in treatment of Type II diabetes mellitus.

21	Idarubicin	Idarubicin; Idarubicin Hydrochloride	-8.1	Intercalates between base pairs and inhibits topoisomerase II activity thereby having antimitotic and cytotoxic activities. Used for treatment of acute myeloid leukemia (AML
22	Canagliflozin	Invokana	-8.0	Inhibitor of (SGLT2) Sodium-glucose co-transporter 2 and an adjunct to diet for improving glycemic control in adult patients of type 2 diabetes mellitus.

Inhibitor lead obtained from previously published reports[18]

Supplementary Table 2: Amino acid residues in active site of PpiB homologues, from other biofilm forming bacteria, that interact with cyclosporine-A or acarbose or dimer of atomic gallium.

Name of the organism	Role in biofilm	GenBank accession number	Homology with PpiB	E-value	Query coverage	Binding Pocket Residues of Cyclosporin	Binding Pocket Residues of Acarbose	Binding Pocket Residues of dimer of atomic gallium[29]
Mycobacterium tuberculosis	(Present study)	AL123456	NA	NA	NA	Arg184	Pro162	Gly203
Staphylococcus aureus	Cystic Fibrosis, Pacemakers, Prosthetic heart valves, Contact Lenses, Orthopaedic implants	WP_061736025.1	35%	9e-20	54%	His186, His58, Pro184, Asp187, Lys183, Val160, Arg59, Leu185, Val178, Lys177	Ala2, Asn3, Tyr4, Pro5, Gln6, Leu7, Gly14, Glu15, Ile16, Pro33, Asn34, Pro37, Lys38, Glu41, Tyr82	Met76, Gly77, Gly78,
Staphylococcus epidermidis	Prosthetic heart valves, Wounds,	WP_049374178.1	33%	8e-18	53%	Tyr186, Asp187, Val160, Pro184, His58, Val185, Arg59, Val178, Ile61, Lys177	Lys9, Asn14, Ile16, Lys17, Lys30, Phe32, Pro33, Asp34, Glu197	Gly75, Gln114
Staphylococcus intermedius	Wounds	WP_019168288.1	31%	3e-19	54%	Asp187, Tyr186, Pro184, His58, Met185, Arg59,	Met1, Thr2, Tyr4, Gln6, Leu7, Lys9,	Gly75, Met76

						Val178, Val60, Lys177	Gln12, Glu13, Pro37, Lys39, Gln41, Tyr82, Glu87	
Streptococcus mutans	Dental biofilm, Orthopaedic implants, Wounds, Prosthetic heart valves, Pacemakers	<u>WP_019320573.1</u>	33%	1e-22	56%	Gln238, Lys240, Lys236, Thr104, Ser234, His106, Gly235, Gln233, Asn232, Pro119, Lys120, Gly121, Gln171, Arg107	Leu5, Val8, Leu9, Phe12, Lys41, Lys43, Leu44, Lys45, Gln46, Leu47, Glu63, Ala64, Leu81, Lys82, Pro85, Val88, Glu89, Leu92, Asp251	Gly123
Staphylococcus saprophyticus	Contact lenses	<u>WP_048792681.1</u>	31%	2e-18	53%	His58, His186, Asp187, Pro184, Leu185, Arg59, Val60, Val178, Lys177	Asn3, Tyr4, Pro5, Gln6, Leu7, Ile16, Lys17, Lys30, Leu31, Leu32, Pro33, Asp34, Val35, Glu93, Gln162, Asp197	Gly75, Gln114
Streptococcus constellatus	Dental biofilm	<u>WP_006270079.1</u>	32%	1e-19	52%	Ala374, His424,	Leu140, Pro141,	Gln335

						Asn373, Gly343, Met344, His326, Met450, Asp451, Lys452, Thr342, Arg327	Val178, Arg179, Trp180, Glu218, Leu219, Gly235, Ile236, Ser237, His238, Lys239, Lys242	
Pseudomonas aeruginosa	Cystic Fibrosis, Wounds, Contact Lenses, Orthopaedic implants, Breast implants,	CRQ97127.1	38%	1e-21	45%	Arg46, Gly141, Leu139, Asp142, His45, Phe44, Glu140, Val47, Gly131, Ile48, Asp132	Lys22, Ala23, Pro24, Leu25, Glu71, Asp72, Glu73, Lys74, Phe115	Thr63

175 NA: not applicable
